# Attenuated *Mycobacterium tuberculosis* SO2 Vaccine Candidate Is Unable to Induce Cell Death

**DOI:** 10.1371/journal.pone.0045213

**Published:** 2012-09-19

**Authors:** Adriana Aporta, Ainhoa Arbues, Juan I. Aguilo, Marta Monzon, Juan J. Badiola, Alba de Martino, Nadia Ferrer, Dessislava Marinova, Alberto Anel, Carlos Martin, Julian Pardo

**Affiliations:** 1 Grupo Apoptosis, Inmunidad y Cáncer, Dpto. Bioquímica y Biología Molecular y Celular, Fac. Ciencias, Universidad de Zaragoza, Zaragoza, Spain; 2 Grupo de Genética de Micobacterias, Dpto. Microbiología, Medicina Preventiva y Salud Pública, Fac. Medicina, Universidad de Zaragoza, Zaragoza, Spain; 3 CIBER Enfermedades respiratorias, Instituto de Salud Carlos III, Madrid, Spain; 4 Research Centre for Encephalopathies and Transmissible Emerging Diseases, Universidad de Zaragoza, Zaragoza, Spain; 5 Unidad Anatomía Patológica, IIS Aragón, Zaragoza, Spain; 6 Servicio de Microbiología, Hospital Universitario Miguel Servet, IIS Aragón, Zaragoza, Spain; 7 Fundación Aragón I+D (ARAID), Gobierno de Aragón, Zaragoza, Spain; 8 Instituto de Nanociencia de Aragón (INA), Universidad de Zaragoza, Zaragoza, Spain; 9 Grupo Inmunidad Celular Efectora (ICE), Dpto. Bioquímica y Biología Molecular y Celular, Fac. Ciencias, Universidad de Zaragoza/IIS Aragón, Zaragoza, Spain; Institut de Pharmacologie et de Biologie Structurale, France

## Abstract

It has been proposed that *Mycobacterium tuberculosis* virulent strains inhibit apoptosis and trigger cell death by necrosis of host macrophages to evade innate immunity, while non-virulent strains induce typical apoptosis activating a protective host response. As part of the characterization of a novel tuberculosis vaccine candidate, the *M. tuberculosis phoP* mutant SO2, we sought to evaluate its potential to induce host cell death. The parental *M. tuberculosis* MT103 strain and the current vaccine against tuberculosis Bacillus Calmette-Guérin (BCG) were used as comparators in mouse models *in vitro* and *in vivo*. Our data reveal that attenuated SO2 was unable to induce apoptotic events neither in mouse macrophages *in vitro* nor during lung infection *in vivo*. In contrast, virulent MT103 triggers typical apoptotic events with phosphatidylserine exposure, caspase-3 activation and nuclear condensation and fragmentation. BCG strain behaved like SO2 and did not induce apoptosis. A clonogenic survival assay confirmed that viability of BCG- or SO2-infected macrophages was unaffected. Our results discard apoptosis as the protective mechanism induced by SO2 vaccine and provide evidence for positive correlation between classical apoptosis induction and virulent strains, suggesting apoptosis as a possible virulence determinant during *M. tuberculosis* infection.

## Introduction

*Mycobacterium tuberculosis* is a facultative intracellular pathogen that infects primarily alveolar macrophages to replicate and disseminate within its host [1]. Hosts and pathogens including *M. tuberculosis* have co-evolved complex mechanisms promoting pathogen elimination or persistence. The major mechanism used by the host to eliminate invading microbes is the immune system, which is the main target used by the pathogen to promote its survival [2]. *M. tuberculosis* has also developed a number of strategies to impair host immune responses, and vaccines against tuberculosis (TB) infection should arm the host to overcome such evasion strategies [3].

Apoptosis is a physiological way of cell death by which multicellular organisms control homeostasis, cell transformation and intracellular infection. This process has been shown to be crucial in the early control of obligate intracellular pathogens such as viruses [2] and some bacteria like *Chlamydia* [4]. In most cases, however, apoptosis is used as a virulence mechanism during the course of infections with facultative intracellular pathogens like *Yersinia, Shigella*, or *Salmonella* [5]–[7]. Despite the fact that *M. tuberculosis* is a facultative intracellular pathogen that shares similar host cells (macrophages), apoptosis has been proposed as a host-mechanism to control infection during experimental TB [8]. It has been suggested that virulent *M. tuberculosis* inhibits apoptosis and triggers necrosis of host macrophages to evade innate immunity and delay the initiation of adaptive immune responses [9]. This suggestion is derived from *in vitro* studies showing that macrophages infected with virulent strains of *M. tuberculosis* undergo an atypical form of cell death sharing characteristics of both apoptotic and necrotic cell death [10], [11]. In contrast to virulent strains, attenuated strains like BCG have shown to induce higher levels of cell death and apoptosis [12]–[15] and this has been proposed as a mechanism that may contribute to host protection during immunization [8], [16]. Controversially, independent groups have shown virulent strains of *M. tuberculosis* to trigger higher levels of cell death by apoptosis than BCG or non-virulent strains [17]–[22]. At present, it is not clear to what extent apoptosis induced by *M. tuberculosis* is a virulence mechanism to promote host colonization or a host mechanism to block bacteria replication.

SO2 is a live attenuated *M. tuberculosis* strain based on inactivation of the *phoP* gene in the clinical isolate MT103 [23]. The *phoP* gene encodes the transcription factor of the two-component system PhoP-PhoR essential for *M. tuberculosis* virulence [24]. SO2 has been shown to be at least as attenuated as the current TB vaccine Bacille Calmette-Guerin (BCG) and to confer protective immunity against pulmonary disease following *M. tuberculosis* challenge in different animal models from mouse to non-human primates [25]–[27]. The rigorous data to date provide robust evidence that SO2 is a promising vaccine prototype with potential to replace BCG.

Apoptosis has been proposed as a mechanism of host-protection against TB [8], [9]. In the present work, we look into a possible role for apoptosis induction as a mechanism to explain the attenuated phenotype of SO2. To this end, we systematically studied the capacity of attenuated SO2 vaccine strain to induce cell death, in parallel with the virulent *M. tuberculosis* MT103 strain and the current vaccine BCG.

## Results

### Attenuated SO2 Strain does not Induce Neither Phosphatidylserine (PS) Translocation Nor Membrane Permeabilization in Primary Macrophages

**Figure 1:**
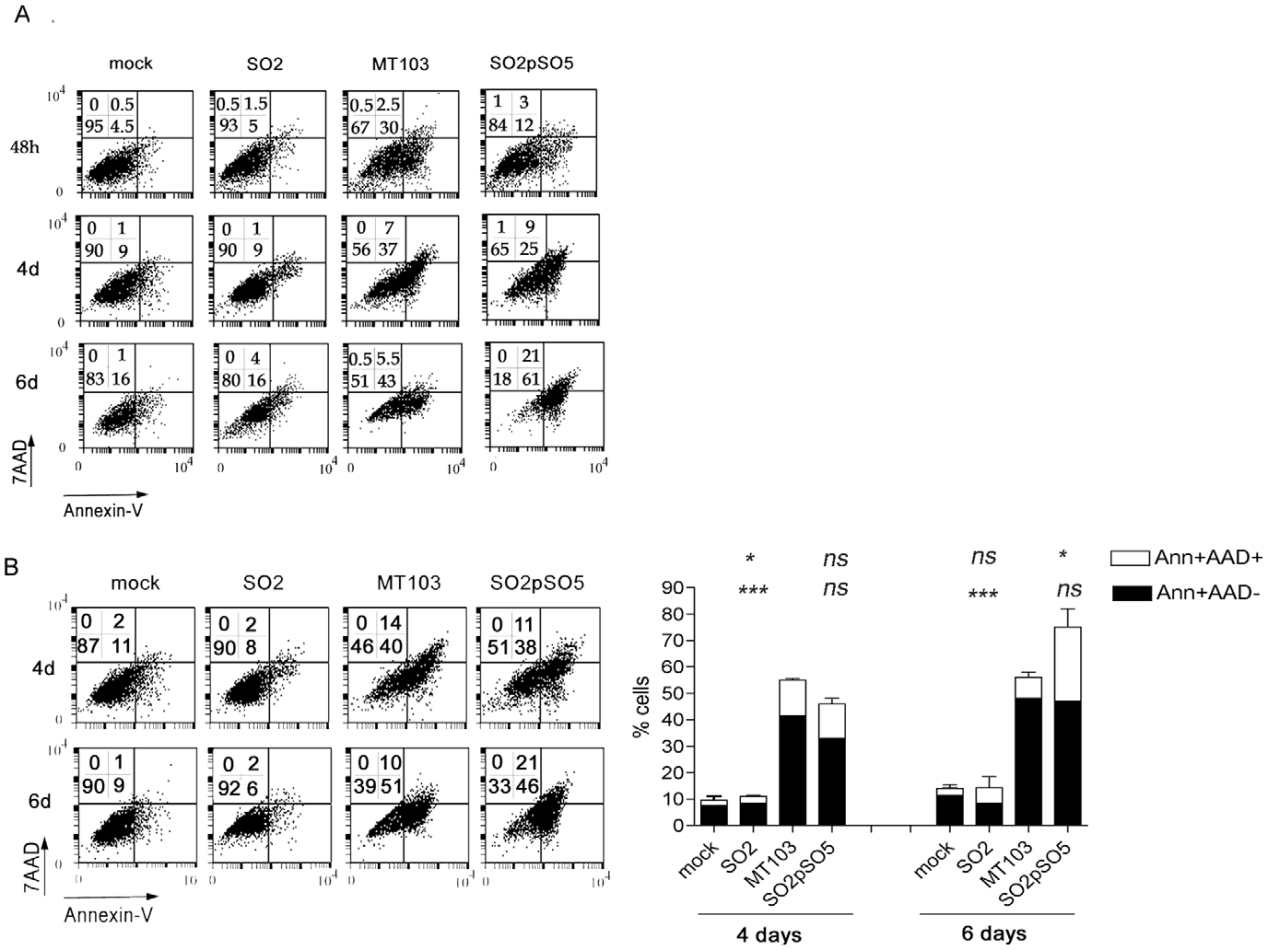
Attenuated SO2 strain does not induce PS translocation in primary mouse macrophages. Mouse bone marrow-derived primary macrophages (BMDM) were mock-treated or infected at low MOI of 1∶1 (A) or 10∶1 (B) with the indicated strains. After 4 hours of infection, cells were washed and incubated with complete medium. At the indicated times post infection, both detached and adhered cells were pooled and PS exposure (annexin-V-FITC) and 7-AAD staining were analyzed by flow cytometry as described in Materials and Methods (A, B). Numbers in the dot-plot diagrams correspond to the percentage (%) of cells in each quadrant. A representative experiment is shown in the left panels. Data in the graph (figure B, right panel) is represented as mean±S.E.M. of at least three independent experiments. Statistical analyses were done with one-way ANOVA with Tukeýs post-test comparing every strain with MT103. Upper symbols  =  statistical analyses of Ann+AAD+ cells; lower symbols =  statistical analyses of Ann+AAD- cells. ns =  not statistically significant; *, **, ***  =  statistically significant; * p<0,05; ** p<0,01; *** p<0,001.

Several works suggest differences in cell death induction on host cells between virulent and non-virulent *M. tuberculosis* strains at low MOI [10], [12], [14], [15]. The apoptotic potential of SO2 compared to MT103 strain was evaluated in primary mouse bone marrow-derived macrophages (BMDM), comparing apoptosis induced on host cells at MOI 1∶1 and 10∶1 (Figure 1). Apoptosis was measured with Annexin V, to analyze phosphatidylserine translocation to the outer leaflet of the plasma membrane, and 7-actinomycin D (AAD) to evaluate plasma membrane integrity. The attenuated SO2 vaccine did not induce cell death at either MOI 1∶1 (Figure 1A) or 10∶1 (Figure 1B). Notably, SO2 was not able to induce cell death even after long incubation times (up to 6 days). In contrast, MT103 induced cell death at both MOI 1∶1 or 10∶1.

At MOI 1∶1 (Figure 1A) a major population of AnnexinV^+^AAD^−^ cells was found after 2, 4 and 6 days of infection. At all times tested, the amount of double-positive AnnexinV^+^AAD^+^ cells, which corresponds to necrotic-like phenotype, was markedly lower (43% AnnexinV^+^AAD^−^ vs. 5.5% AnnexinV^+^AAD^+^ at day 6). A similar result was observed at MOI 10∶1 (Figure 1B), with the majority of dead cells presenting apoptotic phenotype at 4 and 6 days post infection. These results indicate phosphatidylserine translocation and intact membrane integrity, a classical apoptotic phenotype.

We also tested whether complementation of SO2 mutant with the wild-type *phoP* gene would recover the ability to induce apoptosis. As shown in Figure 1A and 1B, the complementation of SO2 with *phoP* (SO2pSO5), which had been reported to restore virulence [23], was also able to restore apoptosis induction capacity at both MOI tested.

Differences observed between virulent MT103 and attenuated SO2 strains are not due to a lower ability of SO2 to infect BMDM, since the entry was not significantly different between both strains (data not shown).The same result was obtained in infection experiments with the GFP-expressing strains, observing a similar initial number of GFP-positive macrophages for both strains (data not shown). It has been previously described that SO2 presents a higher capacity of adhesion to host cell [28]. Our results however, indicate that higher adhesion to plasma membrane does not necessarily leads to an increment of the bacterial entry within the cell. As described previously, SO2 does not replicate in BMDM, in contrast, virulent MT103 and complemented SO2pSO5 strains replicated intracellularly showing approximately a 5-fold increment after 4 days of infection [23].

### Attenuated BCG or SO2 Strains do not Induce PS Translocation, Caspase-3 Activation or Nuclear Fragmentation and Condensation in J774 Macrophages

**Figure 2:**
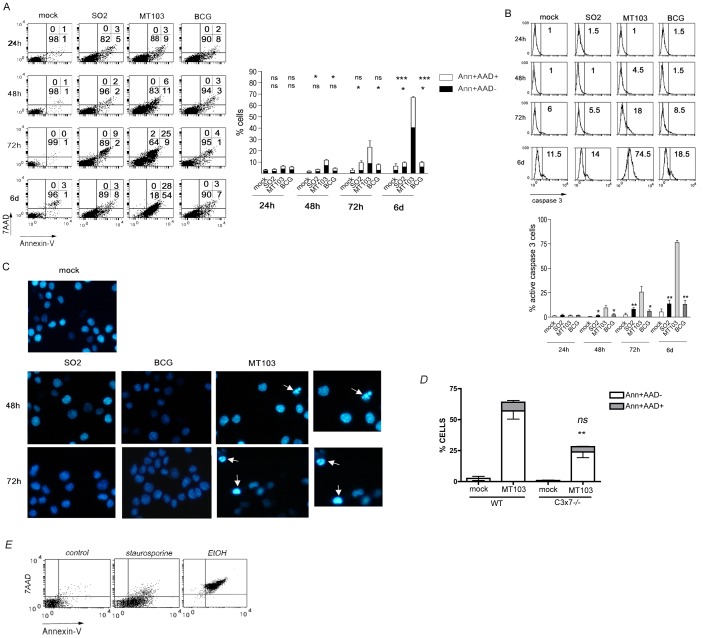
Attenuated SO2 and BCG strains do not induce PS translocation, caspase-3 activation and nuclear apoptosis in J774 mouse macrophages. The mouse macrophage cell line J774 was mock-treated or infected at low MOI (up to 10∶1) with the indicated strains (A-C). Mouse embryonic fibroblasts (MEF) from wild type (WT) of caspase-3 and 7 deficient (c3x7) mice were infected with MT103 (D). After 4 hours, cells were washed and incubated with complete medium. At the indicated times post infection, both detached and adhered cells were pooled and PS exposure on plasma membrane (annexin-V-FITC) and 7-AAD uptake (A, D) or caspase-3 activation (B) were analyzed by flow cytometry as described in Materials and Methods. Numbers in the dot-plot diagrams correspond to the percentage (%) of cells in each quadrant. A representative experiment is shown in the left panels. Data in the graphs (right panels) are represented as mean±S.E.M. of at least three independent experiments. Statistical analyses were done with one-way ANOVA with Tukeýs post-test comparing BCG and SO2 strains with MT103 (A) or MEF.WT with MEF.C3x7^−/−^ (D). Upper symbols  =  statistical analyses of Ann+AAD+ cells; lower symbols =  statistical analyses of Ann+AAD- cells. ns =  not statistically significant; *, **, ***  =  statistically significant; * p<0,05; ** p<0,01; *** p<0,001. Cells were stained with Hoetchs33258 and observed by fluorescence microscopy (C). Pictures from representative fields are shown. Original magnification: x400. Magnifications from the squared sections are shown on the right side. Arrows indicate cells with nuclear apoptotic morphology. (E) J774 cells were treated with 100 nM staurosporine for 24 h or 10% (v/v) ethanol for 90 min. Subsequently, both detached and adhered cells were pooled and PS exposure on plasma membrane (annexin-V-FITC) and 7-AAD uptake was analysed by flow cytometry.

We evaluated the apoptotic potential of MT103 and attenuated SO2 or BCG strains in mouse macrophage cell line J774 at low MOI (up to 10∶1) (Figure 2). We evaluated several markers of classical apoptosis: PS translocation (AnnexinV) and membrane integrity (AAD), caspase-3 activation and nuclear fragmentation and condensation. Only in cells infected with virulent MT103, PS translocation was detected after 48 h reaching a high proportion of apoptotic-like cells after 6 days (54% AnnexinV^+^AAD^−^ vs. 28% AnnexinV^+^AAD^+^) (Figure 2A). Neither SO2 nor BCG strains were able to induce apoptosis on J774 cells even after 6 days of infection (Figure 2A). A similar result was found in the mouse alveolar macrophage cell line MH-S (data not shown). Apoptotic phenotype was also observed when we infected macrophages at low MOI with virulent *M. tuberculosis* H37Rv and the Beijing strain GC1237 (data not shown), suggesting that ability to induce apoptosis is a common feature of virulent *M. tuberculosis* strains. Staurosporine and ethanol were used as controls of dead cells with apoptotic (AnnexinV^+^AAD^−^) or necrotic phenotype (AnnexinV^+^AAD^+^), respectively (Figure 2E).

Next we analyzed if earlier apoptotic markers, such as caspase-3 activation or later features like nuclear fragmentation and DNA condensation, were also induced by MT103 in J774 cells. Intracellular staining of active caspase-3 showed that only the MT103 strain induced caspase-3 activation, with a 74.5% of caspase-3-activated cells at 6 days post infection (Figure 2B). Neither SO2 nor BCG induce caspase-3 activation at any of the time points tested. In addition, caspase-3 enzyme activity was analyzed with a specific chromogeneic substrate (see materials and methods), corroborating results obtained by intracellular staining (data not shown). A typical nuclear apoptotic morphology including nuclear condensation and fragmentation was detected in cells infected with MT103, but not with BCG or SO2 strains (Figure 2C). These data confirm that the virulent MT103 induces a classical apoptotic phenotype unlike the attenuated strains studied.

To test the contribution of caspases to PS translocation and cell death, we infected MEF cells deficient in executioner caspases 3 and 7 (MEF C3x7^−/−^). It is important to remark that both caspases 3 and 7 must be deleted to achieve optimal apoptosis resistance [29]. As it is seen in Figure 2D, MEF C3x7^−/−^ were much more resistant to MT103-induced apoptosis than wild type (WT) cells indicating a critical role for these molecules during apoptosis induced by virulent MT103 strain. We confirmed previously that caspase-3 was activated in MT103-infected MEF cells (data not shown).

In Figure S1, we show that apoptosis induced by MT103 was accompanied by phagocytosis of dying cells, as seen by the presence of orange/yellow cell debris (from cells previously infected with MT103 strain) in the cytosol of fresh green macrophages. This indicates that “eat-me” signals expressed in the surface of apoptotic bodies are intact.

### Attenuated Strains Neither Kill Nor Affect Macrophage Proliferation at Very High MOI

**Figure 3:**
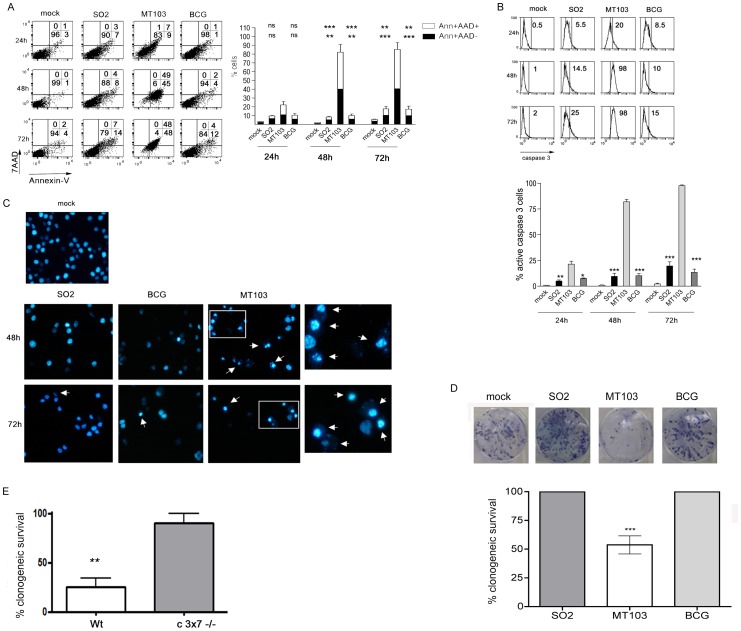
Attenuated BCG or SO2 strains do not kill J774 macrophages at high MOI. The mouse macrophage cell line J774 was mock-treated or infected at high MOI (100∶1) with the indicated strains (A-D). After 4 hours, cells were washed and incubated with complete medium. At the indicated times post infection, both detached and adhered cells were pooled and PS exposure on plasma membrane (annexin-V-FITC) and 7-AAD uptake (A) or caspase-3 activation (B) were analyzed by flow cytometry as described in Materials and Methods. Numbers in each dot-plot diagram correspond to the percentage (%) of cells in each quadrant. A representative experiment is shown in the left panels. Data in the graphs (right panels) are represented as mean±S.E.M. of at least three independent experiments. Statistical analyses were done with one-way ANOVA with Tukeýs post-test comparing BCG and SO2 strains with MT103. Upper symbols  =  statistical analyses of Ann+AAD+ cells; lower symbols =  statistical analyses of Ann+AAD- cells. ns =  not statistically significant; *, **, ***  =  statistically significant; * p<0,05; ** p<0,01; *** p<0,001. Cells were stained with Hoetchs33258 and observed by fluorescence microscopy (C). Pictures from representative fields are shown. Original magnification: x400. Magnifications from the squared sections are shown on the right side. Arrows indicate cells with nuclear apoptotic morphology. For clonogenic survival assay J774 cells were mock-treated or infected at high MOI (100∶1) with MT103, BCG or SO2 strains (D). 4 hours after infection, cells were washed, trypsinized and counted. 150 cells per well were seeded in triplicates (6-well plate) and incubated in fresh medium during 8 days. In the case of MEF.wt and MEF.c3x7−/− cells (E), they were infected with MT103 for seven days. After infection, cells were washed, trypsinized and counted. 150 cells per well were seeded in triplicates (6-well plate) and incubated in fresh medium for eight additional days. After incubation, cells were stained with Crystal Violet and colonies were counted as described in Materials and Methods. Images from single wells of a representative experiment are shown (D). Survival was calculated as percentage of colonies relative to the number of colonies in the non-infected controls (D, E). Values are represented as mean+/− SEM of 4 different experiments. Statistical analyses were done with one-way ANOVA with Tukeýs post-test by comparing MEF.WT with MEF.C3x7^−/−^. ** p<0,01; *** p<0,001.

It is been described that *M. tuberculosis* induces cell death on macrophages at high MOI [11]. To further investigate if BCG and SO2 strains lack the ability to induce apoptosis even at high MOI conditions, we infected J774 cells at MOI 100 (Figure 3). In these conditions, MT103 killed host cells at faster kinetics than observed at the lower MOI used. In addition, most of the cells presented a late apoptotic/necrotic-like phenotype (AnnexinV+7AAD+) at 48 and 72 hours post infection. MT103-infected cells were positive for caspase-3 activation (Figure 3B) and presented condensed nuclei (Figure 3C). Conversely, cells infected SO2 and BCG at high MOI did not show PS translocation, membrane damage, caspase-3 activation or nuclear changes at any time tested (Figure 3 A-C), indicating that these strains are not cytotoxic for host cells under any of the conditions tested.

It should be noted that after 6 days at low MOI (10∶1; Figure 2A) or after 48 h and 72 h at high MOI (100∶1; Figure 3A), a significant % of cells presented membrane damage as stained with AAD. However, it is not possible to differentiate whether these are late apoptotic cells or cells killed by necrosis. The data concerning caspase-3 activation suggest that AnnexinV^+^AAD^+^ cells may correspond to late apoptotic cells. Anyway cells infected with SO2 or with BCG strain did not show any sign of early apoptosis or late apoptosis/necrosis at any time or MOI tested. We have shown that vaccine strains SO2 and BCG do not induce cell death on macrophages up to 6 days post infection, but it is plausible that these strains could have some cytotoxic or cytostatic effect on host cells at longer times. The gold standard test to discern if a specific stimulus is able to kill a target cell at long term is the clonogenic survival assay, which is based in testing the ability of treated cells to form colonies [30]. We infected J774 macrophages with SO2, BCG or MT103 strains and measured whether their growth capacity was affected. At 8 days post infection, there were no differences between SO2- or BCG- infected cells and non-infected controls in their ability to form colonies (Figure 3D), indicating that these cells were viable and their proliferation capacity was not affected by these strains. In contrast, this capacity was highly abrogated in MT103 infected macrophages. Infection experiments at high MOI with the GFP-expressing strains indicated that the initial proportion of infected cells were comparable (between 60%–80%) for the three strains tested (data not shown). This confirms that the differences between MT103 and the attenuated strains were not due to a discrepancy in bacterial entry. Moreover, the initial percentage of MT103-infected cells was comparable with the decrease in the proportion of colonies recovered after infection. Given the low number of cells seeded per well (150 cells in a 9.6-cm^2^ surface), cell-to-cell contact is not expected to occur, suggesting that MT103 was killing all the cells initially infected. Finally, clonogenic survival assay was used to corroborate that MEF.C3×7^−/−^, resistant to MT103-induced apoptosis (Figure 2D), were able to grow to the same extent as the uninfected controls (Figure 3E).

### Virulent *M. tuberculosis*, but not Attenuated Strains, Induces Apoptosis *In Vivo*

**Figure 4:**
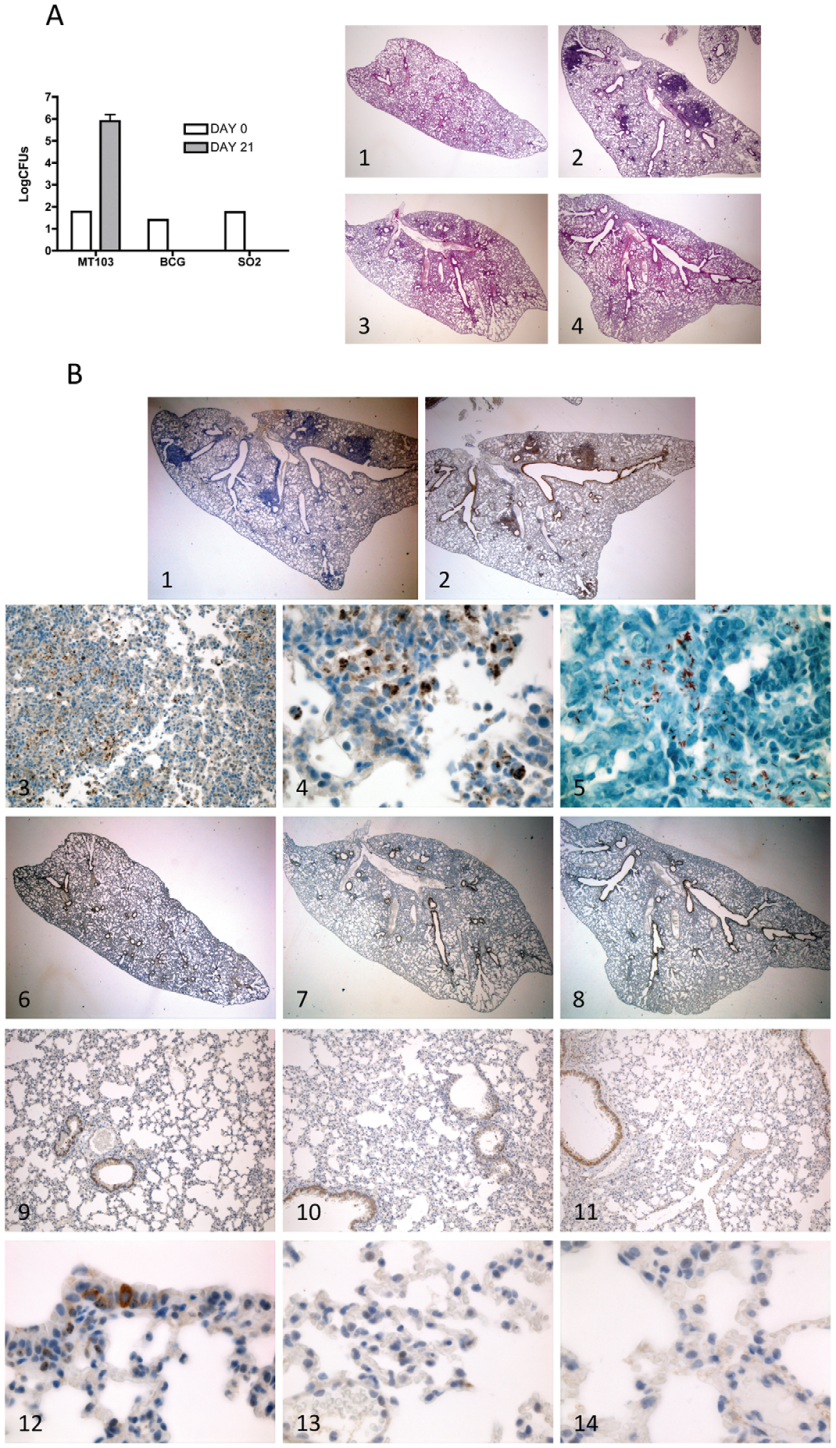
Virulent MT103 strain, but not the attenuated SO2 and BCG strains, replicates in vivo, causes lung pathology and induces apoptosis in mouse lungs. Groups of five C57BL/6 mice were intratracheally infected with a low dose (100 bacteria/mouse) of MT103, BCG or SO2 strains as described in Materials and Methods. Lungs were harvested and CFU counted at 21 days post-infection (A, left panel). Lung histopathology (hematoxilin/eosin) representative images (10x magnification) of mock-treated (A, right panel, image 1) or MT103-, BCG- or SO2-infected mice (A, right panel, images 2, 3, 4, respectively) at 3 weeks post inoculation. Representative images of active caspase 3 immunohistochemical and Ziehl-Neelsen staining of mock-treated or MT103-, BCG- or SO2-infected lungs at three weeks post inoculation (B): primary antibody control of MT103-infected lung section incubated only with secondary antibody (10x magnification) (image 1); active caspase-3 staining of MT103-infected lung section (10x magnification) (image 2); active caspase-3 staining of MT103-infected lung section (100x magnification) (image 3); active caspase-3 staining of MT103-infected lung section (600x magnification) (image 4); Ziehl-Neelsen staining of MT103-infected lung section (600x magnification) (image 5); active caspase-3 staining of mock-treated lung section (10x, 100x and 600x magnification, images 6, 9 and 12, respectively); active caspase-3 staining of BCG-infected lung section (10x, 100x and 600x magnification, images 7, 10 and 13, respectively); active caspase-3 staining of SO2-infected lung section (10x, 100x and 600x magnification, images 8, 11 and 14, respectively).

We have shown that virulent MT103 strain, but not BCG and SO2, replicates intracellularly and induces apoptotic-like cell death in primary and cell line macrophages *in vitro*. To further evaluate these results *in vivo*, we analyzed bacterial replication, lung pathology and active caspase-3 staining in infected lungs from C57BL/6 mice intratracheally inoculated with a low dose (100 CFU) of MT103, BCG and SO2. In agreement with previous published data [26], CFU counts in lungs showed that only MT103 was able to replicate in lungs three weeks post infection (Figure 4A, left panel). In addition, lung pathology examination showed the presence of multifocal inflammatory sites and areas of necrotic tissue only in mice infected with MT103 (Figure 4A, right panel, image 2), but not with BCG or SO2 (Figure 4A, right panel, images 3 and 4). F4/80 staining showed a high presence of macrophages in the inflammatory aggregates (data not shown). Finally, active caspase-3 staining clearly demonstrated high degree of apoptotic cells in all the inflammatory foci distributed across the lung parenchyma in MT103-infected lungs (Figure 4B, images 2, 3, 4) as well as the presence of bacilli by Ziehl-Neelsen staining (Figure 4B, image 5). Lungs of BCG- and SO2-inoculated mice were negative for caspase-3 staining (Figure 4B, images 7, 8), and did not show inflammatory foci, macrophage infiltration or Ziehl-Neelsen positive staining.

## Discussion

Rigorous preclinical studies for protection and immunogenicity in relevant animal models have shown that *phoP*-deficient SO2 strain represents a promising candidate to prevent TB [25]–[27] Recently, a mechanism for the improved immunity afforded by SO2 has been described [31]. Vaccination with SO2 induced enhanced antigen-specific memory T cell response in mice. As apoptosis has been proposed as a mechanism of host-protection against TB [17], we questioned the possible role of apoptosis induction contributing to enhance SO2-induced immunity.

Our results show that SO2 and current vaccine BCG, did not kill host cells *in vivo*, and *in vitro* at any MOI tested, and did not affect macrophage proliferative capacity as assessed by clonogenic survival assay at the highest MOI. Conversely, *M. tuberculosis* MT103 virulent strain induces classical apoptosis on host cells both *in vivo* and *in vitro*.

It has been described that virulent strains of *M. tuberculosis* induce high levels of apoptosis on infected lungs in both humans and mice [12], [32]–[35]. *M. tuberculosis* is an intracellular pathogen that has acquired different strategies to maintain an intracellular lifestyle. As a result, apoptosis induction should be an advantage when compared to necrosis: 1) plasma membrane integrity is not affected during apoptosis, allowing bacteria to elude extracellular defenses by maintaining an intracellular environment; 2) apoptotic cells are quickly cleared by other macrophages, promoting an effective infection mechanism to colonize new cells [34].

Unlike MT103, attenuated SO2 strain presents limited replication in macrophages [23] **.** Results may suggest that apoptosis induced by MT103 could be due to high bacterial burden reached during replication, in agreement with previous data [36]. Trying to discern this question, we conducted the high MOI (100∶1) experiments with SO2, with the aim to reach a high initial bacterial load, to reflect the situation observed with MT103 after bacterial replication at low MOI. We have two major experimental findings to indicate that cell death is not just a function of bacterial numbers:

1. Infection of macrophages with non-virulent strains at very high initial MOIs (100 and 300∶1) does not kill cells even after 6 days of infection, suggesting that high bacterial burden does not necessarily trigger cell death.
2. While MT103, SO2 and BCG replicate to the same extend in fibroblast cells [37], only the virulent MT103 induces cell death.

This data provide compelling evidence that intracellular replication does not necessarily have to conduct to host cell death.

Here we show that, in addition to other apoptotic signature markers, caspase-3 is activated both *in vivo* and *in vitro* and presents enzymatic activity during cell death induced by virulent MT103. Supporting our results, other works also demonstrate that this protease is activated during cell death induced by virulent strains [19]. Moreover, it has been recently shown that granulomas from tuberculosis patients present high levels of active caspase-3 staining [33]. Caspase-3 activity has been shown to be crucial for PS translocation and apoptotic nuclear morphology [38]. Our data show that caspase-3 activity is associated with PS translocation and nuclear changes induced by MT103 and suggest that macrophages undergo classical apoptosis during infection. Indeed, typical nuclear apoptotic phenotypes have been previously shown [36]. In order to analyze the role of effector caspases during *M. tuberculosis*-induced apoptosis in host cells, we conducted macrophage infection experiments in the presence of pan-caspase inhibitors. However, we were unable to observe any inhibitory effect (data not shown). It is not clear whether caspase inhibitors are effective in macrophages, as they have been previously described to enhance lipopolysaccharide-induced apoptosis in this cell type [39], [40]. Validating the efficacy of pan-caspase inhibitors, we did not observe apoptosis inhibition on macrophages treated with staurosporine, a classical apoptotic stimulus (data not shown). Further supporting importance of caspases during MT103-induced apoptosis, mouse embryonic fibroblasts from caspase-3 and −7 deficient mice showed a marked resistance to apoptosis induced by MT103 strain, suggesting a role for caspase-dependent cell death. Supporting our conclusions it was previously shown that a *M. tuberculosis* mutant that did not express ESAT6 was not able to induce caspase-3 mediated apoptosis [41]. A mechanism that links ESAT6 and caspase-3 activation has been recently proposed by Grover et al. ESAT-6-induced apoptosis was shown to be dependent upon the cleavage of the macrophage molecule BAT-3 by caspase-3 [42]. These findings help to explain why the SO2 strain that, as discussed below does not secrete ESAT6, fails to induce caspase-3 activation and any type of cell death.

Our findings demonstrate that host cells infected with virulent strain MT103 at low MOI resulted positive for apoptotic signature markers. These results are sustained by several independent groups [17]–[22]. Other groups have suggested that virulent *M. tuberculosis* strains inhibit macrophage apoptosis and promote necrosis, as a potential mechanism of spreading infection [9], [10], [13]–[16], [43]–[47].

Trying to understand these apparent discrepant results, a plausible scenario could be that at early stages of infection, when MOI in the lungs should be very low, virulent *M. tuberculosis* infects alveolar macrophages and kill them by apoptosis. This would allow the recruitment of fresh macrophages that recognize and phagocytose apoptotic bodies containing the viable mycobacteria, permitting intracellular replication in the absence of a potentially hostile extracellular environment. Thus *Mycobacterium marinum* induces macrophage apoptosis in granulomas to attract and infect new host cells and spread infection in zebra fish [34]. At advanced stages of the pulmonary disease, when bacterial burden is high, virulent bacteria would lyse host cells to reach the extracellular environment and be transmitted to new hosts. Lee *et al* reported that macrophages loaded with a high bacterial number rapidly undergo cytolysis [36]. Correlating with these results, we show at high MOI that most cells infected with MT103 lose their plasma membrane integrity, which corresponds to necrotic-like phenotype.

Our findings indicate that *phoP* plays a central role in MT103-induced host cell death. We show that *phoP* complementation clearly recovers capacity of SO2 to induce apoptosis on primary macrophages at low MOI. Other authors have shown that at high MOI *phoP*-mutant strains are unable to kill host cells [11]. Consistent with this data, the *phoP*-mutant SO2 strain does not induce cell death at high doses. Being a promising TB vaccine candidate, this data could serve to explain the sound attenuation profile of SO2, demonstrated previously [25], [26].

The expression of *NuoG* by mycobacteria has been associated with inhibition of apoptosis on host cells [48]. As PhoP is controlling *NuoG* expression [*24*], there existed the possibility that SO2 was promoting apoptosis on infected cells. However, our results clearly show that SO2 is not able to induce apoptosis, despite *NuoG* downregulation, indicating that other factors are involved in the modulation of the apoptotic machinery. Our results and other works strongly indicates that ESAT-6, whose expression is restricted to virulent strains [49], is a good candidate to mediate *M. tuberculosis*-induced cell death [22], [41], [42], [50]–[52]. Its depletion reduces apoptosis induced by H37Rv [22], [41], [42]. Indeed, the non-apoptogenic BCG and SO2 strains do not produce (BCG) or secrete (SO2) ESAT-6. BCG lacks the RD1 region containing the *esat6* gene, while this protein is produced but not secreted in SO2, as PhoP positively regulates this process [24], [53].

It is been recently shown that *M. tuberculosis* is able to disrupt the phagosome and translocate to the cytosol by a mechanism dependent on ESAT-6. This process seems to be crucial for Mtb to kill the host cell [51]. A previous work shows that SO2 is not able to arrest phagosome maturation, being localized in acid compartments [28]. This indicates that SO2 does not translocate to the cytosol, unlike virulent strains. This is in agreement with the lack of ESAT-6 secretion observed in SO2 and could explain why this strain is not killing the host cell.

The results shown in this work provide evidence for correlation between replication in macrophages and apoptosis induction. Data from our lab showing apoptosis induced on macrophages by other replicative strains (H37Rv or from the Beijing family) support this hypothesis. Several studies have shown impaired survival or killing of intracellular mycobacteria by host cell apoptosis [19], [54]–[56]. However, infection experiments with MT103 show clear correlation between intracellular growth and apoptosis induction on host cells. We further show in vivo that apoptosis induced by MT103 is accompanied by the increase in lung bacterial loads. Conversely, SO2, which does not induce apoptosis, is not able to replicate intracellularly. Altogether, our data suggest that apoptosis on host cells is not a bactericidal mechanism, at least for the strains studied in this work. Far from being an innate protective mechanism for the macrophage, apoptosis induction could be an effective mechanism of spreading the infection into new fresh macrophages. *M. marinum* uses apoptosis induction in granulomas of zebra fish as a Trojan horse to attract and infect new host macrophages [34]. It should be noted that our results and conclusions are mainly based on experiments with macrophages and that different results might be obtained in other cell types susceptible to infection like dendritic cells or neutrophils.

There are many controversies regarding the role of apoptosis during *M. tuberculosis* infection. In some works apoptosis is suggested to be a protective mechanism, as it is inhibited by virulent *M. tuberculosis* strains. Other groups describe apoptosis as a mechanism of virulence. Different *in vitro* experimental models, cell lines and/or protocols could account for such discrepancies. However, analysis of apoptosis in *in vivo* infection models may provide a more realistic scenario, with less experimental variants, and allowing reproducible results between the different groups. Some groups have analysed cell death induced by *M. tuberculosis in vivo* [12], [32]–[35], [16], [45], [46]. However, more data are clearly necessary to reach a consensus about the role of apoptosis during *M. tuberculosis* infection.

In agreement with other authors, we have observed apoptosis induction in mouse *M. tuberculosis*-infected lungs. Our hypothesis is that host macrophage apoptosis could be advantageous for *M. tuberculosis* to promote infection, since it would offer *M. tuberculosis* an inconspicuous hideout to avoid host immune responses, while spreading infection into new host cells.

The rigorous data to date provide robust evidence that SO2 is a promising vaccine prototype with potential to replace BCG. Absence of apoptosis observed during SO2 infection could serve to understand the sound attenuation profile of this vaccine [26].

## Methods

### Cell Culture

Mouse embryonic fibroblasts (MEF) immortalized with SV40 virus from wild type (MEF.WT) (Health Protection Agency, Cat No.98061101) or caspase-3 and 7 KO mice (MEF.C3×7^−/−^; kindly provided by Richard A Flavell) [29], the mouse bone marrow-derived cell line J774 (Health Protection Agency, Cat No.85011428) were cultured at 37°C and 5% CO_2_ in DMEM medium supplemented with 10% inactivated foetal bovine serum (Biological industries) and 2 mM glutamine (Biological industries).

### Mouse Bone Marrow-derived Macrophages (BMDMs)

The protocols for animal handling were previously approved by University of Zaragoza Animal Ethics Committee (protocol number PI03/11). Femurs and tibias were obtained from 6–12 week old C57BL/6 mice. Cells were aseptically eluted from excised bones by vigorously injecting DMEM through the bone marrow cavity. Then, cells were resuspended in DMEM supplemented with 10% inactivated foetal bovine serum (Biological industries), 2 mM glutamine (Biological industries), streptomycin (100 µg/ml) and penicillin (100 U/ml) (GIBCO), and 10% supernatant of L-929 (Health Protection Agency, Cat No.85011425) cell fibroblasts as source of granulocyte/macrophage colony stimulating factor. Mouse bone marrow cells were seeded at a density of 1×10^6^ cells in 6-wells plates and allowed to differentiate for 7 days at 37°C and 5% CO_2_.

### Bacterial Strains and Growth Conditions

*M. bovis* BCG Pasteur 1173P2 [57], *M. tuberculosis* clinical isolate MT103, its *phoP* mutant derivative SO2 (kanamycin resistant), and the *phoP*-complemented SO2 strain (SO2pSO5) [23], were used in this study. The *M. bovis* BCG and *M. tuberculosis* wild type and mutant strains were rendered fluorescent by the transfer of plasmid pMV361H *gfp* (Green Fluorescent Protein) [58]. Mycobacteria were grown at 37°C in Middlebrook 7H9 broth (BD Biosciences) supplemented with 0.05% Tween 80 and 10% Middlebrook albumin dextrose catalase enrichment (ADC; BD Biosciences) and, when required, the medium was supplemented with 20 µg/ml of kanamycin or hygromycin. Before use, fresh mycobacterial cultures were centrifuged at 20 g for 5 min to remove bacterial clumps. The virulence and quality of our stocks is always confirmed *in vivo* in mice. All virulent stocks used to perform these experiments showed high virulence *in vivo*.

### Cell Infection with *M. tuberculosis*

Cells were seeded in wells or flasks (depending on the experiment; Sarsted) and allowed to attach to the plastic overnight. Bacterial suspension for infection was prepared from an exponential phase culture. Culture was centrifuged at low speed to eliminate clumps. Bacterial concentration was determined by measuring optical density at 600 nm. For infection, the medium was removed and replaced with bacterial suspension of each strain in DMEM containing the number of CFU/ml required to obtain a multiplicity of infection (MOI) of 1∶1, 10∶1 or 100∶1 bacteria per macrophage. After 4 h at 37°C, the medium was removed and cells were washed three times with PBS to remove extracellular bacteria (reduction to a non-significant number of extracellular bacilli). MOIs were confirmed by plating serial dilutions of each bacterial suspension on solid Middlebrook 7H10 medium supplemented (BD Biosciences) with 10% Middlebrook ADC enrichment (BD Biosciences). In the mock controls cells were treated in the same way except that no bacteria were added to cell cultures.

### Clonogenic Assay

Cells (5×10^5^) per well were seeded in 6-well plates and infected with the different *M. tuberculosis* strains at high MOI (100∶1). After 4 hours in macrophages, and 7 days in MEFs, cells were trypsinized and 150 cells were seeded per well in a final volume of 3 ml in a 6-well plate. Cells were then allowed to grow during 8 days at 37°C. After that time, medium was removed and cell colonies were counted after fixing and dying them for 20 min with a mixture of glutaraldehyde (6.0% v/v) and crystal violet (0.5% w/v) at room temperature.

### Apoptosis Analysis *In Vitro*

Cells (1.5×10^6^) were seeded in 25 cm^2^ flask and infected the following day with the different strains as explained before. After 4 hours-infection, cells were washed and fresh medium was added and cells were incubated at 37°C. At different time points, both supernatant and trypsinized cells were collected together and 5×10^5^ cells were used to analyze apoptosis by Flow Cytometry flow cytometry or fluorescence microscopy to analyze nuclear morphology as follows:

Phosphatidylserine (PS) exposure and membrane integrity were analyzed by using Annexin-V and 7-actinomycinD (BD Biosciences) and flow cytometry according to manufacturer instructions. Briefly, cells were washed with PBS and incubated with Annexin-V and 7AAD in Annexin-binding buffer for 15 min. After that, cells were washed twice with PBS, fixed with 4% paraformaldehyde (PFA) during 30 min and washed again with PBS. Both PBS and PFA solutions contained CaCl_2_ 2.5 mM.

Caspase-3 activation was analyzed by using an antibody against active caspase-3 (BD Biosciences) and flow cytometry as previously described [59].

Caspase-3 enzymatic activity was analysed at different times after infection. Cells were collected and lysed in a buffer containing 1% Triton X-100. Serial dilutions (log 2) of cell lysates (starting at 1×10^6^ cell equivalents) were incubated at 37°C with 200 µM caspase-3 substrate Ac-DEVD-pNA (ENZO Life sciences) in the following reaction buffer: 100 mM HEPES pH 7.5, 20% (v/v) glycerol, 20 mM DTT and 0.5 mM EDTA. Absorbance at 405 nm was measured in a UV-visible absorbance spectrophotometer microplate reader at different time points (t = 0, 30 min, 1 h, 2 h, 4 h and 16 h).

Nuclear morphology was analyzed by fluorescence microscopy with Hoechst 33342. Cells were fixed with 4% PFA for 30 min, washed with PBS and mounted on slides over 3 µl of Fluoromount-G (Southern biotech) containing 10 µg/ml Hoechst 33342 (Invitrogen). Images were taken using a fluorescence microscope (E600/E400, Nikon) equipped with digital photograph system (DXM 1200F, Nikon) and analyzed with Nikon ACT-1.

### *M. tuberculosis* Infection *In Vivo* in Mice

The protocols for animal handling were previously approved by University of Zaragoza Animal Ethics Committee (protocol number PI43/10). Intratracheal infection was performed with 100 CFU of bacteria in 50 µl of PBS per mouse. To deliver bacterial suspension, isoflurane anesthetized mice were orally intubated with a lachrymal olive luer-lock (UNIMED), 30 mm in length and 0.6 mm in diameter. Three weeks post-infection, lungs from each animal were harvested and placed in PBS for bacterial burden evaluation or in 10% formaldehyde for histological studies. To analyze bacterial replication, lungs were homogenized using GentleMacs homogeneizer (Miltenyi Biotec) and CFU counted by plating serial dilutions on solid Middlebrook 7H10 medium supplemented (BD Biosciences) with 10% Middlebrook ADC enrichment (BD Biosciences). Day 0 CFUs corresponds to the number of bacteria originally used to infect mice.

For histological and Immunohistochemical studies, lungs were harvested and fixed in 4% Neutral Buffered Formalin, placed in toto into Histology cassettes and processed in the Xpress X50 rapid tissue processor (Sakura, Japan) until paraffin embedding. Paraffin blocks were made and cut at 3 um. Sections were stained with Hematoxylin-Eosin and Ziehl-Nielsen stain methods for histological assessment. For immunohistochemistry, sections were deparaffinized in xylene and hydrated in a gradient alcohol series from 100% to 70% and running water for 5 minutes. Heat mediated antigen retrieval was performed by means of PT-Link (Dako, Denmark) by heating the slides at 92°C in low or high pH buffer (Target Retrieval Solution, High pH or Low pH, Dako, Denmark) depending on the antibody, for 20 min and then washed in wash buffer (Dako, Denmark).

Endogenous peroxidase was quenched (Peroxidase-Blocking Reagent, EnVision™, Dako, Denmark) followed by incubation with Caspase-3 active (R&D systems) and F4/80 (Abcam) primary antibodies. For visualization, Dako EnVision System HRP was used depending on the antibody with a suitable secondary antibody (HRP labeled goat anti rabbit or rabbit anti rat) following suppliers procedure.

The colour reaction was developed by DAB+ chromogen in substrate buffer (Dako, Denmark), resulting in a brown reaction product. Sections were counterstained with Mayer’s hematoxylin, dehydrated in a gradient series of alcohol, cleared in xylene and mounted. In negative controls, the primary antibody was omitted.

For histological analysis, the whole lung of each animal was studied with a Leica DM5000B microscope and representative pictures of each slide taken with a Leica DFC 420C camera at 10x and 40x magnification. Histological findings and positive labeled cells and location compared to negative controls were assessed and recorded.

### Phagocytosis Assay

A detailed description of the phagocytosis assay can be found as Supporting Information (M&M S1).

## Supporting Information

Figure S1 **Apoptotic bodies from MT103 infected macrophages were phagocytosed by fresh macrophages.** J774 macrophages were labelled with Cell Tracker Orange and mock-treated or infected with MT103, BCG or SO2 strains (MOI 100∶1). After infection for 24h, detached and adhered cells were pooled, washed and counted. Cells were added in a ratio of 2∶1 to fresh Cell Tracker Green-labelled macrophages, and incubated for 4 hours. Subsequently, cells were fixed in 4% PFA and mounted on slides with Fluoromount-G. Fluorescence images were taken at room temperature on a confocal microscope (TCS SP2; Leica) using a x60 objective (HCX PL APO CS; Leica), NA 1.25, immersion oil and confocal software (version 2.61; all Leica). Photoshop CS2 software (Adobe) was used for minor adjustments to contrast. Images shown are representative of at least two independent experiments. Green fluorescent cells represent fresh macrophages; red, mock-treated or infected cells; merged (yellow), is indicative of phagocytosed MT103-infected macrophages. Absence of merged fluorescence in mock-treated or BCG- and SO2- infected cells mixed with fresh macrophages indicates that results obtained with MT103 are not due to unspecific staining.
(TIF)

M&M S1 **Phagocytosis.**
(DOC)
