# Supplementary figures and images for: Attenuated Mycobacterium tuberculosis SO2 Vaccine Candidate Is Unable to Induce Cell Death
Source: PLoS One. 2012 Sep 19;7(9):e45213. doi: 10.1371/journal.pone.0045213 (PMC3446966; doi:10.1371/journal.pone.0045213)

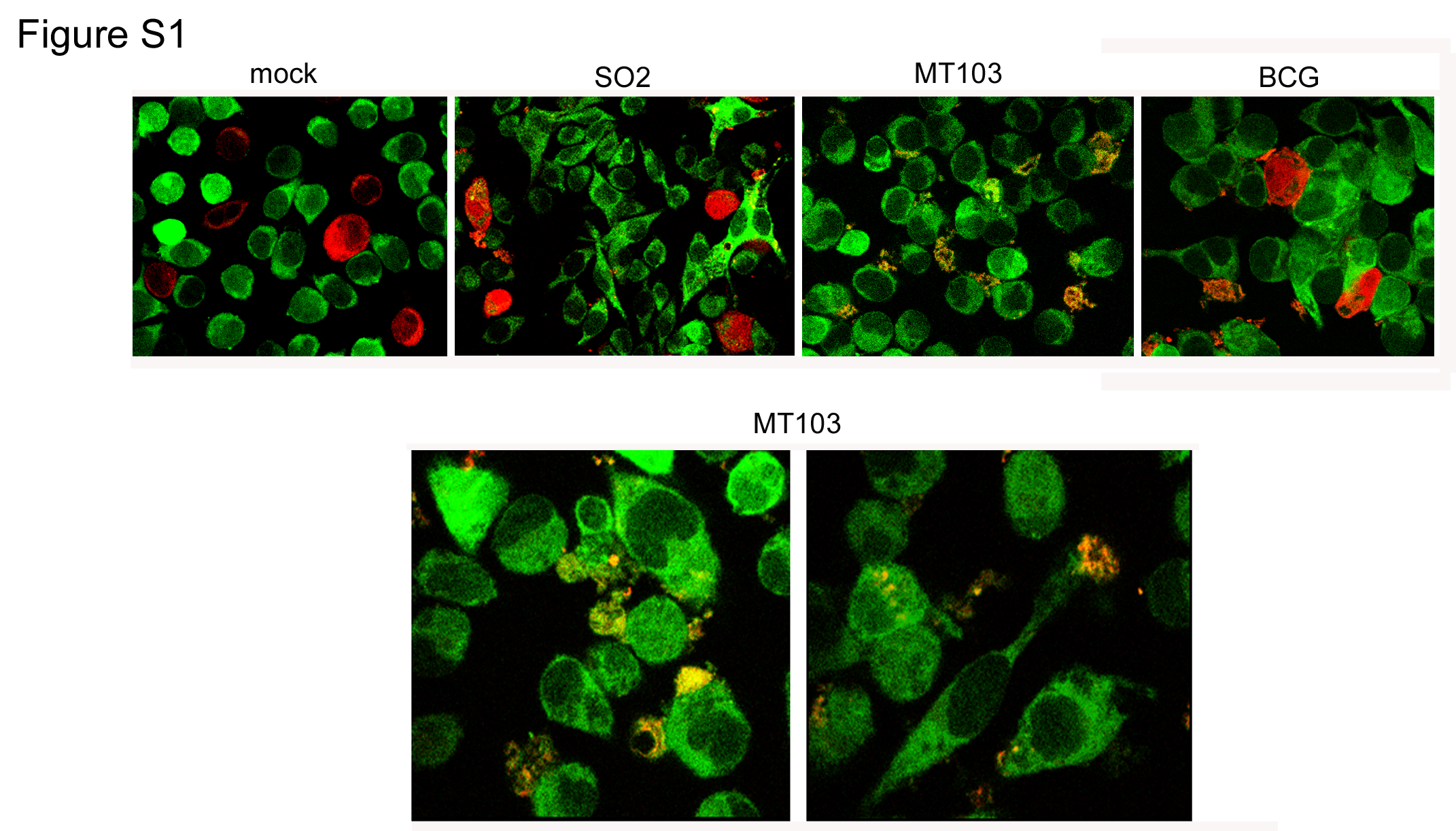

Supplement: Figure S1 — Apoptotic bodies from MT103 infected macrophages were phagocytosed by fresh macrophages. J774 macrophages were labelled with Cell Tracker Orange and mock-treated or infected with MT103, BCG or SO2 strains (MOI 100∶1). After infection for 24h, detached and adhered cells were pooled, washed and counted. Cells were added in a ratio of 2∶1 to fresh Cell Tracker Green-labelled macrophages, and incubated for 4 hours. Subsequently, cells were fixed in 4% PFA and mounted on slides with Fluoromount-G. Fluorescence images were taken at room temperature on a confocal microscope (TCS SP2; Leica) using a x60 objective (HCX PL APO CS; Leica), NA 1.25, immersion oil and confocal software (version 2.61; all Leica). Photoshop CS2 software (Adobe) was used for minor adjustments to contrast. Images shown are representative of at least two independent experiments. Green fluorescent cells represent fresh macrophages; red, mock-treated or infected cells; merged (yellow), is indicative of phagocytosed MT103-infected macrophages. Absence of merged fluorescence in mock-treated or BCG- and SO2- infected cells mixed with fresh macrophages indicates that results obtained with MT103 are not due to unspecific staining. (TIF) [file pone.0045213.s001.tif]
