# Supplementary material for: Attenuated Mycobacterium tuberculosis SO2 Vaccine Candidate Is Unable to Induce Cell Death
Source: PLoS One. 2012 Sep 19;7(9):e45213. doi: 10.1371/journal.pone.0045213 (PMC3446966; doi:10.1371/journal.pone.0045213)
Supplement: M&M S1 — Phagocytosis. (DOC) [file pone.0045213.s002.doc]

*M&M S1. Phagocytosis.*

J774 Cells were incubated with 2 µM Cell Tracker Orange CMRA (Invitrogen) for 30 min at 37ºC. Afterwards, they were centrifuged and washed with DMEM medium with 10% inactivated foetal bovine serum (Biological industries) for 20 min at 37ºC. 5 x 10^5^ cells were seeded per well and allowed to attach for 24 h at 37ºC. Subsequently, they were infected with the different strains (MOI 100:1) as described before and incubated for 48 h at 37ºC.

The following day, fresh J774 cells were stained with 2 µM Cell Tracker Green CMFDA (Invitrogen) following the same steps as explained before and 1 x 10^5^ cells were seeded on glass coverslips in 24-well plates and incubated at 37ºC for 24 h. 48 h after infection, infected macrophages were collected (supernatant and trypsinized fraction pool) and 2 x 10^5^ cells (ratio infected cell: fresh macrophages 2:1) were added to each well containing fresh J774.

Cells were incubated for different time points and after removing supernatant they were fixed with 4% PFA for 30 min, washed with PBS and mounted on slides over 3 μl of Fluoromount-G. Cells were visualised with a Leica SP2 AOBS confocal scanning microscope. Images were collected using the microscope in sequential mode with a 40x oil immersion lens (lens specification, HCXPLAPO NA 1.25; Leica), a line average of 16 and a format of 1024x1024 pixels. The confocal pinhole was 1 Airy unit.
